# Putative Effect of Aquifer Recharge on the Abundance and Taxonomic Composition of Endemic Microbial Communities

**DOI:** 10.1371/journal.pone.0129004

**Published:** 2015-06-17

**Authors:** Renee J. Smith, James S. Paterson, Cally A. Sibley, John L. Hutson, James G. Mitchell

**Affiliations:** 1 School of Biological Sciences, Flinders University, Adelaide, South Australia 5001, Australia; 2 School of the Environment, Flinders University, Adelaide, South Australia 5001, Australia; INRA, FRANCE

## Abstract

Drought events and the overexploitation of freshwater resources have led to the increased need to manage groundwater reserves. Aquifer storage and recovery (ASR), whereby artificial water is injected into aquifers for storage, is one of the proposed methods by which freshwater supplies can be increased. Microbial clogging following injection, however, is a major issue. Here, during laboratory simulations of ASR, we used flow cytometry and bar-coded pyrosequencing to investigate changes in microbial abundance and community dynamics. Bacterial abundance ranged from 5.0 × 10^4^ to 1.4 × 10^7^ cells ml^-1^ before the addition of synthetic wastewater. Following wastewater addition, a 25-fold decrease in abundance was observed, coinciding with a 12-fold increase in viral abundance. Taxa shifted from an overrepresentation of *Sphingomonadales*, *Sphingobacteriales*, *Rhodospirillales*, *Caulobacterales*, *Legionellales*, *Bacillales*, *Fusobacteriales* and *Verrucomicrobiales* prior to the addition of synthetic wastewater to *Burkholderiales*, *Actinomycetales*, *Pseudomonadales*, *Xanthomonadales*, *Rhodobacterales*, *Thizobiales* and *Thiotrichales* following the addition of synthetic wastewater. Furthermore, a significant difference in overall taxonomic composition between the groundwater samples before and after the addition of synthetic wastewater was observed, with water samples exhibiting more similarity to sediment samples after wastewater was added. Collectively, these results suggest that ASR may alter the taxonomic composition of endemic microbial communities and that complete profiles of groundwater properties, including microbial community abundance and composition need to be taken into consideration when selecting aquifers for ASR practices.

## Introduction

More than 97% of the world’s accessible freshwater reserves are found in aquifers [1], making groundwater one of the most important resources on the planet. In the midst of global warming and drought events, the need to manage these freshwater resources is becoming increasingly important [2–4]. Overexploitation of groundwater by domestic, agricultural and industrial practices have led to the wide spread degradation of aquifers [5,6]. Therefore, the augmentation of local groundwater supplies via the introduction of stormwater, reclaimed water and impaired surface water is of growing interest [7,8].

Aquifer storage and recovery (ASR) is one of the proposed methods by which water supplies can be increased throughout the year [4]. ASR involves the injection of artificial recharge into aquifers during periods of excess supply, where it is then stored and subsequently re-used when needed [9]. However, ASR can be difficult to maintain long term, due to the high degree of clogging, which can occur within days of injection [10]. Clogging within the aquifer decreases the flow rate, thus hindering subsequent water extraction [11,12]. Among the three classes of clogging (biological physical and chemical), biological or more specifically, microbial clogging is of particular interest [13].

Artificial recharge commonly contains high concentrations of nitrogen and organic carbon [14]. The high concentrations of these chemicals in the infiltrating water can act as a food source for the native microbial communities [15]. The native microbial communities then undergo growth periods, whereby an accretion of microorganisms form within pore spaces that would otherwise hold water and permit flow [16]. The formation of biofilms within the porous media has been shown to significantly reduce flow rate [13], thus affecting the storage and recovery capabilities of the aquifer.

Many studies have investigated the impact of ASR on groundwater flow rate [10,17–19]. However, to our knowledge, there are no studies that attempt to characterise the microbial communities present. Consequently, there is a lack of information regarding which bacterial species are present in aquifers, and what influence the infiltrate plays on community composition. Bar-coded pyrosequencing [20,21] allows for the characterisation of a relatively large number of individual samples simultaneously, generating detailed phylogenetic and taxonomic information of the microbial communities present [22,23]. Utilizing such methods to comprehensively characterise microbial communities within aquifers undergoing ASR practices will provide the basis for improved operation and design ASR. Thus, the aims of the current study were to investigate microbial community composition in ASR simulations, and to determine the effect of introduced artificial recharge on changes in these communities.

## Materials and Methods

### Site Description

Groundwater samples were sourced from Mackreath Creek Bore A, located within the Scott Creek Catchment, South Australia (35’095S 138’618E) during June 2012. Site access was made available by SA Water through Flinders University. Mackreath Creek catchment is predominantly comprised of native vegetation [24] and this site was chosen as it has not been found to have any contaminating chemicals or other characteristics which would lead to enhanced clogging [24].

### Groundwater sampling

Groundwater was collected from a piezometer which consisted of a 142 mm diameter PVC casing, at a slot depth of 12 m. To ensure the most representative and uniform water sample from the aquifer formation was collected, the bore was first purged of 3 bore volumes, or until environmental parameters had stabilized [25]. Water was obtained using a submersible 12 V, 39 m Monsoon pump (EnviroEquip). A total of 60 L of groundwater was collected for use in the laboratory trial, while 5 L was collected for bar-coded pyrosequencing.

Physical parameters of dissolved oxygen (DO; mg l^-1^), pH, electrical conductivity (EC; mS cm^-1^) and temperature (°C) were measured using a YSI DO meter, YSI flow cell and YSI 556 Multi-Probe System (YSI Inc.). Samples for initial nutrient analysis were stored at 4°C in the dark and analysed within 24 h. Dissolved organic carbon (DOC) and total nitrogen (TN) concentrations were determined in triplicate using the combustion catalytic oxidation methods on a TOC-L Series analyser (Shimadzu). For enumeration of heterotrophic bacteria and virus-like particles (VLP), triplicate 1 mL water samples were fixed with gluteraldehyde (0.5% final concentration) and incubated at 4°C for 15 min, then quick frozen in liquid nitrogen and stored at -80°C prior to flow cytometry analysis [26].

### Column set up and experimentation

**Fig 1:**
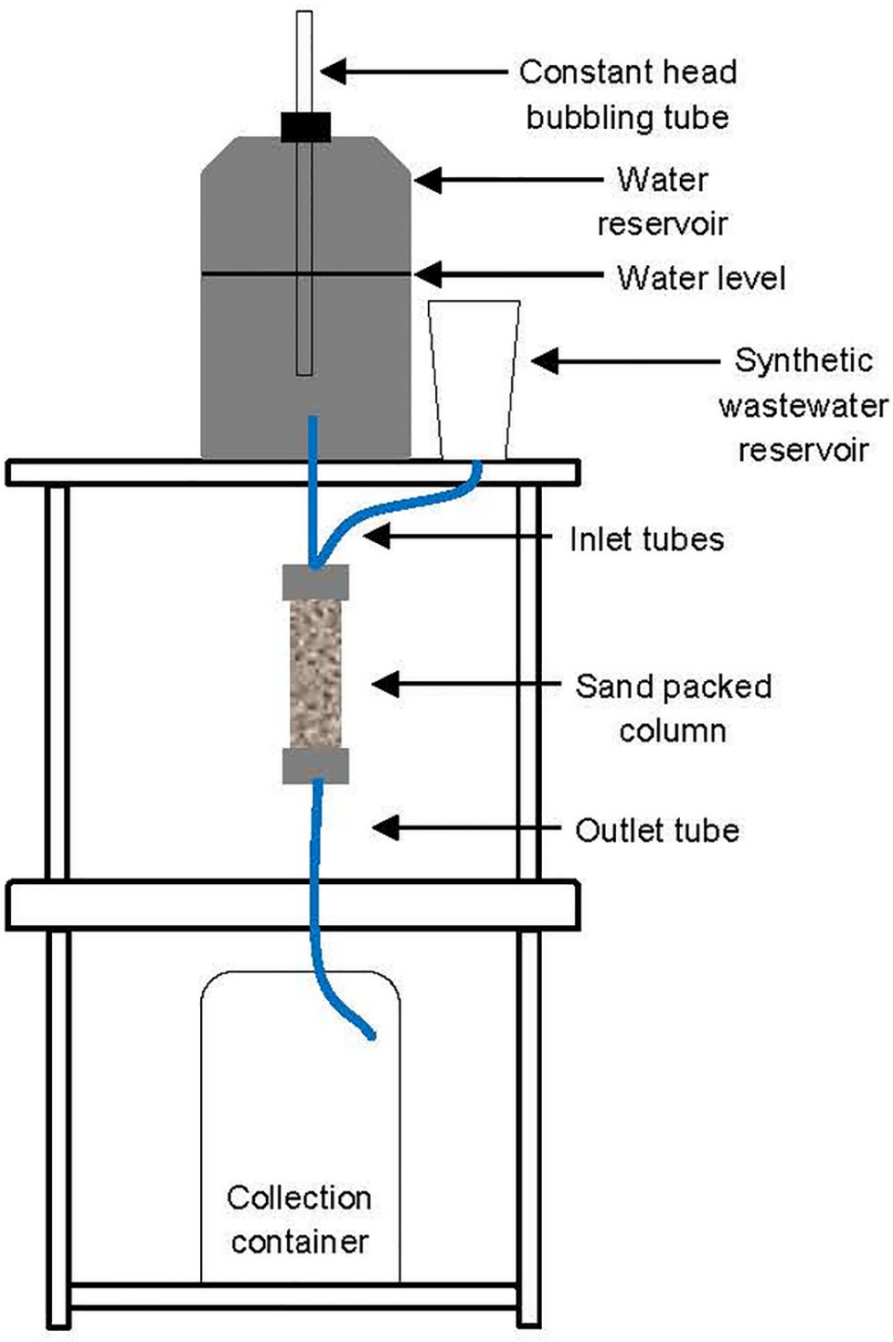
A schematic representation of sediment columns used in the study. Three stainless steel columns, 15 cm in length and 4.5 cm in diameter, were autoclaved and filled with sterile non-reactive silicate fine grain sand, approximately 600 μm in diameter. Each column was connected to a 20 L water reservoir, 30 cm above the columns. Groundwater was gravity fed into the each column for 64 days. Synthetic wastewater was introduced into each column on day 35. Sterile collection containers were located under each column for groundwater recirculation and sample analysis.

A schematic representation of the columns used in this study is shown in Fig 1. To prevent contamination, all glass equipment was autoclaved while plastic equipment was washed with 10% HCL, followed by 0.2 μm filtered MilliQ. The experiment was conducted in a temperature controlled laboratory at 20 ± 2°C, and all columns were kept in the dark to mimic groundwater conditions.

Once assembled, 20 L of groundwater was fed through each column for 64 days. The same groundwater was recirculated over the duration of the study so as to ensure there were no new microbes or nutrients introduced, generating a closed system. Samples for flow cytometric and nutrient analysis were collected at 12 h intervals. DO, pH, EC, and flow rate [27] were also measured every 12 h. At day 34, 5 L of water was collected from each column for barcoded pyrosequencing. On day 35, the second phase of the trial commenced, with the addition of synthetic wastewater, which was gravity fed into each column. The synthetic wastewater used in the experiment contained the following components dissolved in MilliQ water (mg l^-1^); 163.2, milk powder; 16.2, C_12_H_22_O_11_; 37.6, C_2_H_3_NaO_2_; 6, KH_2_PO_4_; 78, (NH_4_)_2_SO_4_; 30, CH_4_N_2_O; 0.1, FeCl_3_; and 1, NaOH [28]. Synthetic wastewater was injected directly into the tubing, 1 cm above the column, to simulate the addition of artificial recharge (Fig 1). Prior to injection, synthetic wastewater was autoclaved to prevent microbial contamination. The columns were run under these conditions for a further 29 days with sample collections every 12 h. Due to low flow rates following the addition of synthetic wastewater, 5 L of water from each column for the purpose of barcoded pyrosequencing was collected from day 56 onwards. After 29 days of injecting synthetic wastewater into the columns, 10 g sediment samples for bar-coded pyrosequencing were collected in sterile conditions at the top, 0 cm, and the bottom, 15 cm of each column.

### Nutrient analyses

From each column at 12 h intervals, 2 x 50 ml samples of water were collected. DOC and TN analysis were conducted in triplicate using the combustion catalytic oxidation methods on a TOC-L Series analyser (Shimadzu).

### Microbial enumeration

Bacteria and VLPs were enumerated using an Accuri C6 flow cytometer (Becton Dickinson), which was fitted with a 488 nm blue laser and green (530/30 nm), orange (585/40 nm) and red (670 nm) filters. Prior to analysis, triplicate samples were thawed and diluted to 1:10 with 0.2 μm filtered TE buffer (10mM Tris, 1 mM EDTA, pH 8). Samples were then stained with SYBR Green-I solution (1:20000 dilution; Molecular Probes, Eugene, OR) and incubated in the dark at 80°C for 10 min [26]. As an internal size and fluorescence standard, 1 μm diameter fluorescent beads (Molecular Probes, Eugene, OR) were added to each samples at a final concentration of approximately 10^5^ beads ml^-1^ [29]. For each sample, forward scatter, side scatter and green (SYBR Green-I) fluorescence were acquired for two minutes. FlowJo (Treestar, Inc.) software was used to analyse data collected from each sample, where differences in cell side scatter and SYBR Green fluorescence were used to discriminate between VLP and bacterial groups [26,30,31].

### Sample filtration, microbial community DNA extraction and sequencing

Following sample collection, water samples for bar-coded pyrosequencing were filtered through 5 μm membranes to remove sediment particles. Microbial biomass was then collected on a 0.22 μm membrane filter. Microbial community DNA from the water samples were extracted using the PowerWater DNA Isolation Kit (MoBio laboratories, Inc., Carlsbad, CA, USA), while the microbial community DNA from the sediment samples were extracted using the PowerMax Soil DNA Isolation Kit (MoBio laboratories, Inc., Carlsbad, CA, USA). DNA concentration and quality were determined using a Qubit fluorometer (Quant-iT dsDNA HS Assay Kit; Invitrogen) and by 1.5% TBE agarose gel electrophoresis (Bioline). High molecular weight DNA was then sent to the Molecular Research LP (MR DNA; Texas, USA) for 16S rRNA gene based bar-coded pyrosequencing [32]. Bacterial diversity of aquifer clogging was analysed by amplification of the 16S rRNA gene using the primers 27F (5’-AGRGTTTGATCMTGGCTCAG -3’) and 519R (5’-GTNTTACNGCGGCKGCTG -3’). Sequencing was conducted on the GS-FLX pyrosequencing platform using Titanium reagents (Roche).

### Data analysis

Euclidean distance matrices were calculated for the square-root transformed data to determine the differences in the geophysical parameters between columns before and after the addition of synthetic wastewater using Primer for Windows (Version 6.1.13, Primer-E, Plymouth; [33]. Significance was then determined using a Permutational Multivariate Analysis of Variance, PERMANOVA + version 1.0.3 add-on to PRIMER [34,35], using 9999 unrestricted permutations.

To determine the overall effect synthetic wastewater had on the microbial cell abundance, rank-abundance analysis was used to identify the degree of structure within a distribution. The abundance of bacterial and VLP abundances were ranked from 1 to 100 and then plotted on a log-log graph of mean abundance versus rank. Power-law trend lines were calculated with the random noise, represented by the characteristic downward roll-off, omitted [36,37].

Unassembled DNA sequences were processed using Quantitative Insights Into Microbial Ecology (QIIME) as previously described [38]. The quality filtering criteria were a minimum quality score of 25, minimum 200 bp in length, no ambiguous bases and no mismatches in the primer sequence. The remaining sequences were clustered into operational taxonomic units (OTUs) based on sequence similarity using UCLUST v1.2.21q [39], with a minimum identity of 97%. Taxonomic assignments were made at different phylogenetic levels using the RDP naïve Bayesian rRNA Classifier [40], with 80% taxonomic confidence and an E-value of 0.001.

To compare the relative levels of bacterial OTU diversity within the groundwater samples, rarefaction and Alpha diversity statistics including library coverage and phylogenetic diversity, Chao 1 [41], were calculated for each sample using QIIME [38]. Bray-Curtis similarity distance matrices were calculated for square-root transformed data to test the difference between groundwater samples before and after synthetic wastewater addition, using Primer 6 for Windows (Version 6.1.13, Primer-E. Plymouth; [33]. Canonical analysis of principal coordinates (CAP) [34] on the sum of squared canonical correlations was used to determine whether there were any significant differences between microbial community composition before and after synthetic wastewater addition, as well as between the water and sediment samples. The a priori hypothesis that the taxonomic composition was different between the groups were different was tested in CAP by obtaining a *P-*value using 9999 permutations. CAP ordinations were generated using order level classifications.

Where significant difference were found using CAP analysis, the percent contribution of each taxa to the separation between groundwater before and after synthetic wastewater addition were assessed using similarity percentage (SIMPER) analysis [42]. Comparison of water samples at day 64 to sediment samples from within the columns were carried out to distinguish potential biofilm forming taxa. The resulting top 90 percent of all taxa were used to determine the shifts in taxonomy between the groups. To determine those taxa that were consistently contributing to the overall dissimilarity between the groundwater samples before and after synthetic wastewater addition, the ratio of the average dissimilarity to standard deviation (Diss/SD) was used. A Diss/SD ratio of greater than 1.4 was used to indicate key discriminating taxa [43].

## Results

### Overview of the biogeochemical conditions, hydraulic conductivity and microbial abundance in column experiments

**Fig 2:**
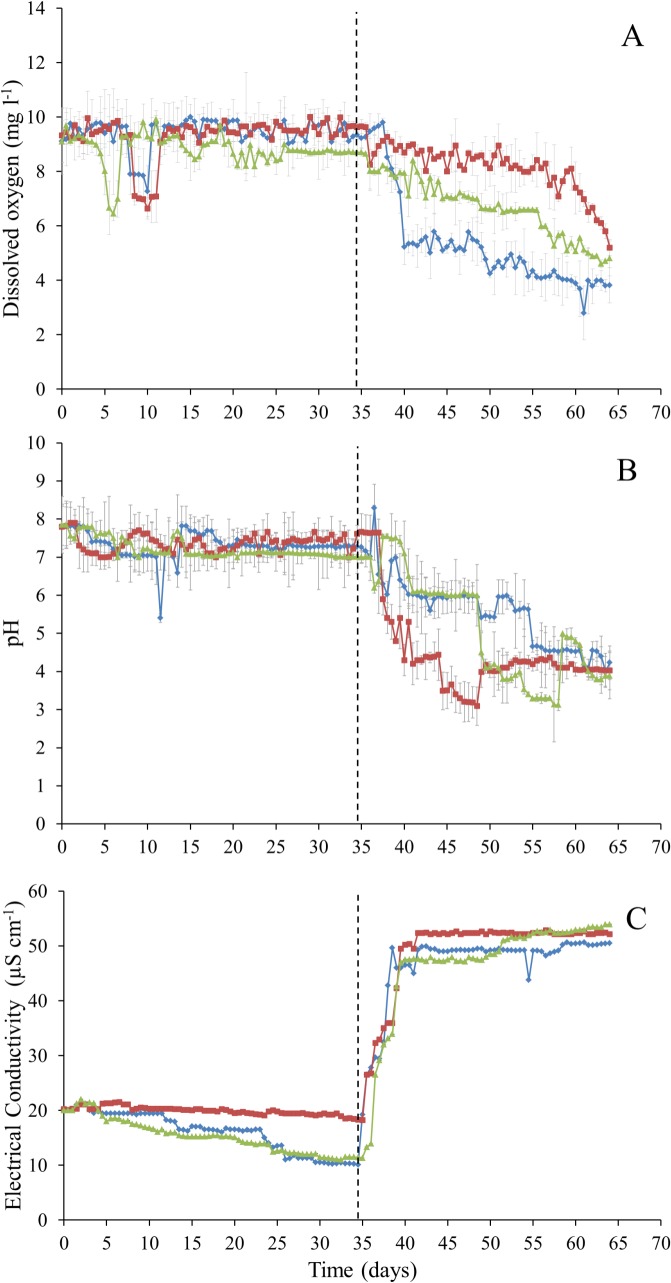
Environmental parameters collected from column water samples every 12 h over a 64 day period. (A) Dissolved oxygen concentrations (DO, mg l^-1^) (B) pH (C) Electrical conductivity (EC, μS cm^-1^). Error bars represent ± standard error. Dashed line represents commencement of synthetic wastewater addition.

**Fig 3:**
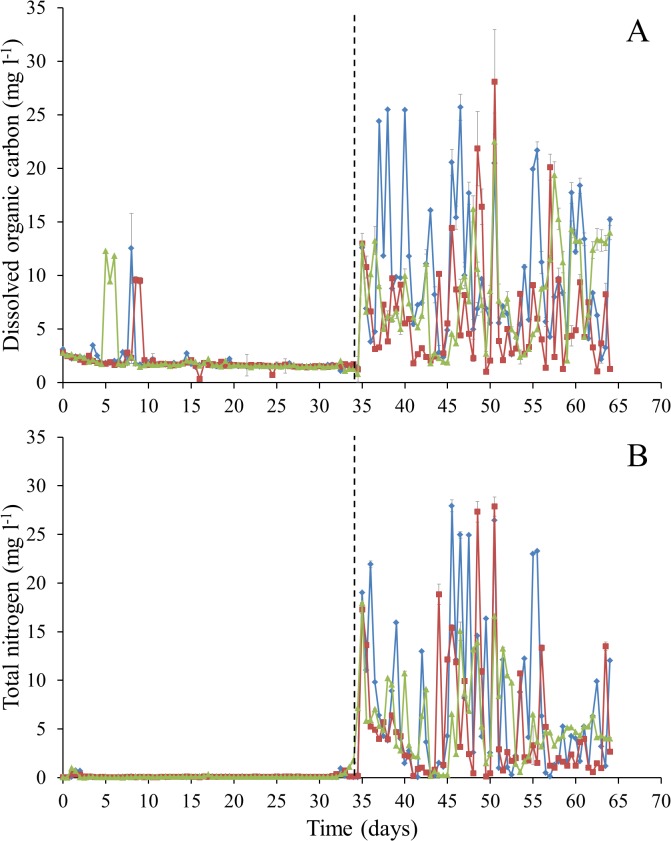
Nutrient analysis from column water samples every 12 h over a 64 day period. (A) Dissolved organic carbon (DOC, mg l^-1^) (B) Total nitrogen (TN, mg l^-1^). Error bars represent ± standard error. Dashed line represents commencement of synthetic wastewater addition.

**Fig 4:**
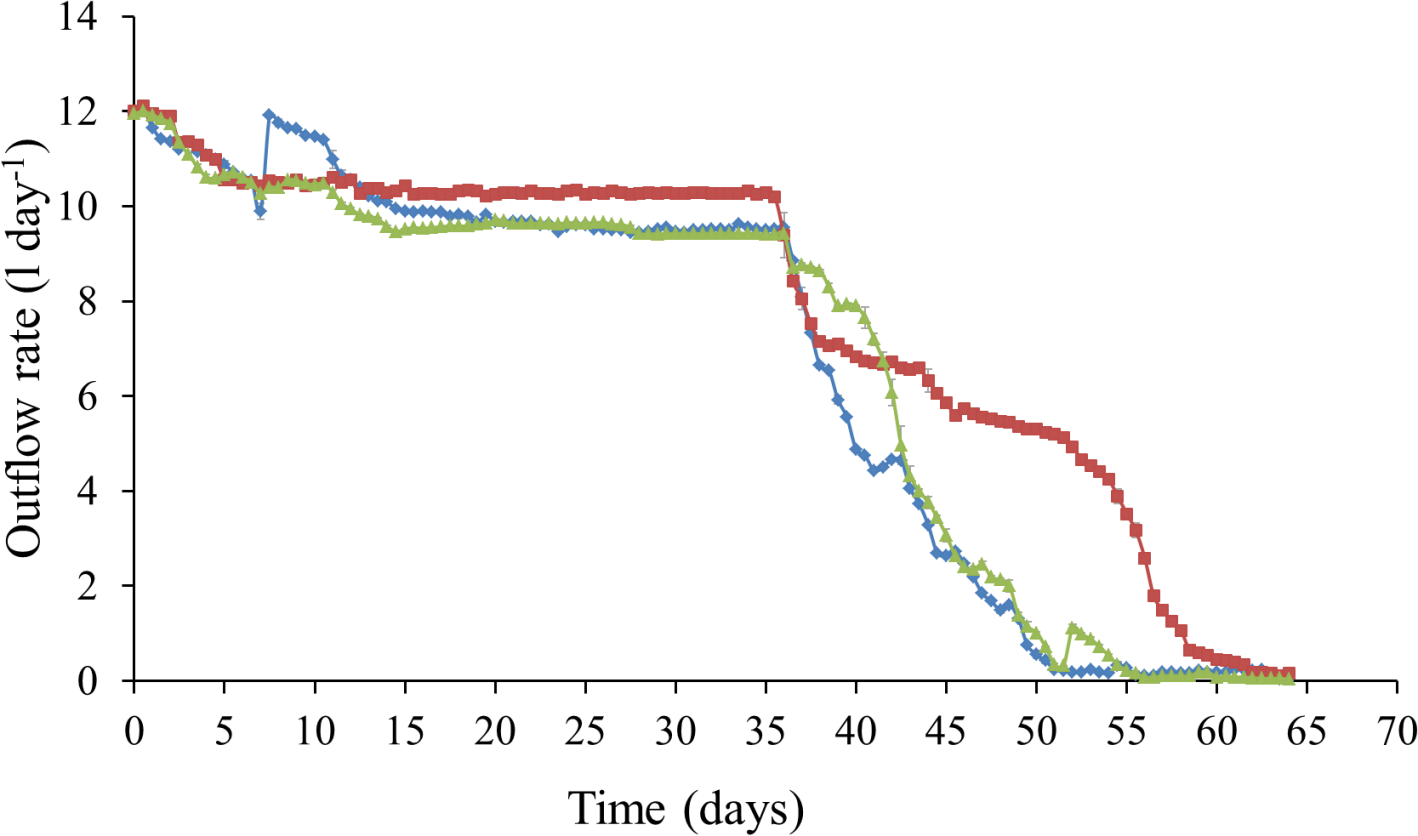
Outflow rate (l day^-1^) from column water samples every 12 h over a 64 day period. Error bars represent ± standard error. Dashed line represents commencement of synthetic wastewater addition.

PERMANOVA analysis exhibited a significant difference between all biotic, physical and chemical variables before and after the addition of synthetic wastewater (*P*-value = 0.0001). More specifically, following the addition of synthetic wastewater, DO concentrations decreased from approximately 9.2 to 4.6 mg l^-1^ from day 35 to day 64 (Fig 2A). There was also a decrease in pH, whereby at day 35, pH in all 3 columns ranged from 7–7.6, however at day 64 pH ranged from 3.9–4.2 (Fig 2B). Nutrient concentrations for DOC and TN became more variable following the addition of synthetic wastewater (Fig 3). Flow rates showed a marked decrease from 9.42–10.24 l day^-1^ at day 35 to 0.03–0.16 l day^-1^ at day 64 (Fig 4).

**Fig 5:**
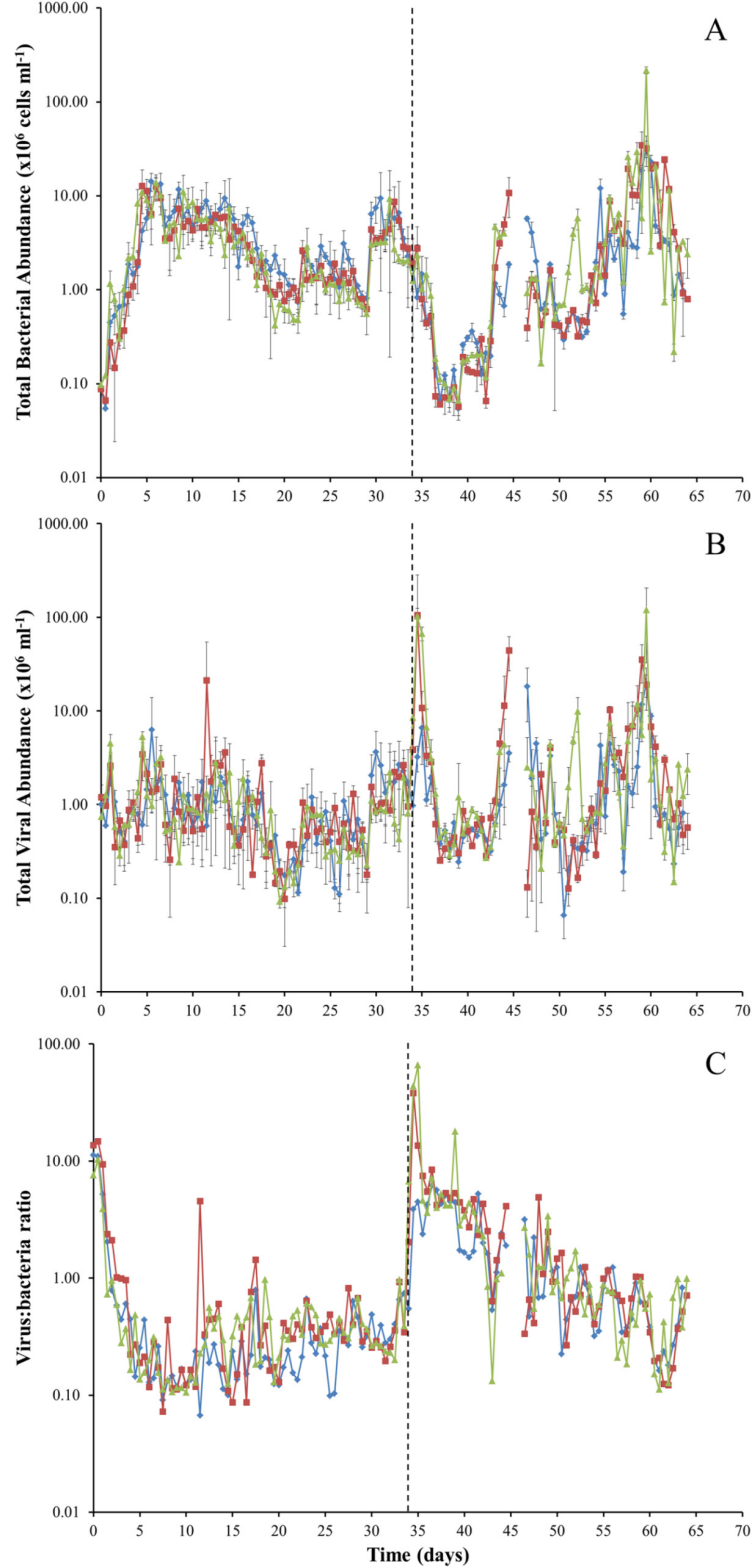
Mean (A) bacterial and (B) VLP abundance (cells ml^-1^) and (C) Virus: bacteria ratio from column water samples collected every 12 hours over 64 days. Errors bars represent ± standard error. Dashed line represents commencement of synthetic wastewater addition.

Mean bacterial abundance ranged from 5.4 × 10^4^–1.4 × 10^7^ cells ml^-1^ before the addition of synthetic wastewater and from 5.4 × 10^4^–2.2 × 10^8^ cells ml^-1^ after wastewater addition (Fig 5A). Mean VLP abundance ranged from 9.2 × 10^4^–2.1 ×10^7^ cells ml^-1^ and from 6.6 × 10^4^–1.2 × 10^8^ cells ml^-1^ before and after synthetic wastewater addition, respectively (Fig 5B). Initial virus:bacteria ratio (VBR) values ranged from 7.6–14.7 at the beginning of the experiment then decreased to 0.1 to 1.0 up to day 34. Following the addition of wastewater (day 35) VBR values increased up to 65.9 then decreased to day 64 (Fig 5C). Rank abundance analysis of bacterial and VLP abundances revealed a power law distribution, whereby an the exponent became more negative following the wastewater addition (S1 Fig).

### Bar-coded pyrosequencing

Following the removal of reads less than 200 bases and filtering by base quality, a total of 237,613 sequences were obtained from the groundwater and column experiments (http://dx.doi.org/10.7910/DVN/29510). Within these sequences, groundwater, prior to its addition to the columns, was dominated by Actinobacteria and Proteobacteria accounting for 60.2% and 30.5%, respectively (S2 Fig and S1 Table). Within these phyla, *Actinomycetales* and *Burkholderiales* contributed to 59.7% and 16.1%, respectively (S2 Table.). After the addition of synthetic wastewater at day 64 a shift was seen in the groundwater within the columns, whereby columns 1, 2 and 3 were dominated by Proteobacteria and Bacteroidetes, contributing to between 30.5–52.6% and 35.3–45.5%, respectively (S2 Fig and S1 Table.). A shift was also present at the order level after 34 days, whereby Proteobacteria were dominated by *Sphingomonadales* accounting for between 18.0–37.8%. Bacteroidetes were dominated by *Sphingobacteriales* accounting for between 35.0–45.4% of the 3 columns (S2 Table.).

Following the addition of nutrients at day 64, groundwater within the columns was dominated by *Burkholderiales* contributing to between 34.3–45.9% of the order level diversity (S2 Table.). Sediment samples within the columns at day 64 were also dominated by Proteobacterial groups at 0 cm and 15 cm (S2 Fig and S1 Table.). However, at 0 cm this phylum was dominated by *Burkholderiales*, contributing to between 24.0–58.9%, while at 15 cm *Rhodocyclales* dominated, accounting for between 30.8 and 52.4% of the diversity within the 3 columns (S2 Table.).

Chao1 rarefaction curves exhibited clear differences between groundwater samples following the introduction of synthetic wastewater at day 64, with OTU richness highest in the water samples following the addition of synthetic wastewater (S3A Fig). Sediment at the top of the columns where the nutrients were introduced had the highest overall OTU richness (S3B Fig). Furthermore, despite extensive sequencing of the microbial communities within the columns, none of the accumulation curves reached an asymptote (S3 Fig).

**Fig 6:**
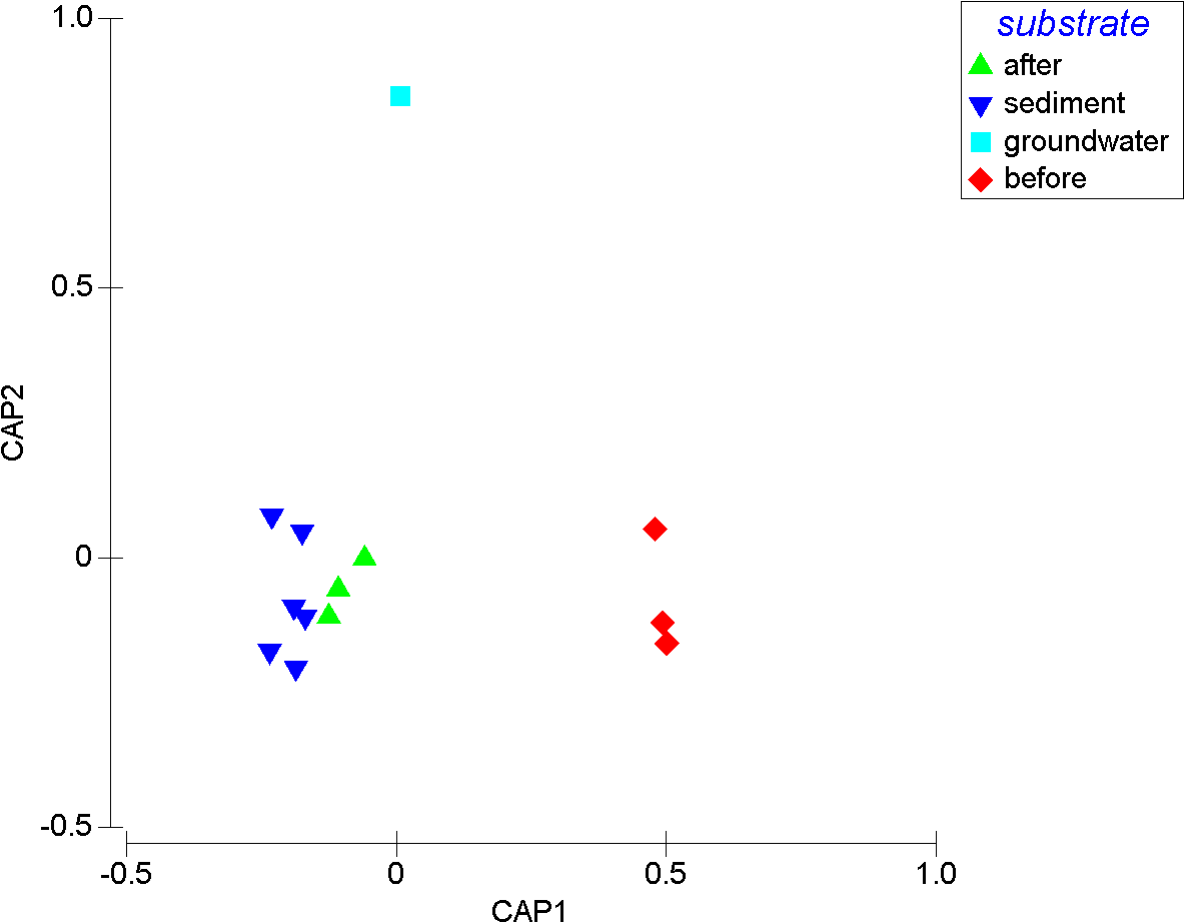
Taxonomic comparison of bacterial communities within the column experiments. CAP analysis (using m = 7 principle coordinate axes) is derived from the sum of squared correlations of DNA fragments matching the RDP database, order level (E-value E<1x10^-3^). Significance P = 0.01 and the first axis explained = 0.95 of the total variation.

To determine the overall impact of nutrient addition to the groundwater microbial community's structural composition, the original groundwater was compared to water before and after the addition of nutrients as well as the sediment samples at the top and bottom of the columns. CAP ordination revealed a clear separation of data, whereby the water after the addition of nutrients, clustered closer to sediment samples at the top and bottom of the column and not to the water samples before the addition of nutrients (Fig 6); (P = 0.01) (S3 Table.). Furthermore, a strong association between the multivariate data and the hypothesis of taxonomic differences was seen, indicated by the large size of their canonical correlations (δ^2^ = 0.99). Cross validation of the CAP model showed 100% of samples were correctly classified (S3 Table.).

SIMPER analysis revealed the main distinguishing taxa associated with the groundwater samples before the addition of synthetic wastewater were *Sphingomonadales*, *Sphingobacteriales*, *Acidobacteriales*, *Rhodospirillales*, *Legionellales*, *Spartobacteriales*, *Syntrophobacterales*, *Bacillales*, and *Verrucomicrobiales*, collectively accounting for 35% of the dissimilarity (S4 Table.). Alternatively, the main distinguishing taxa associated with the groundwater after the addition of synthetic wastewater were *Burkholderiales*, *Flavobacteriales*, *Pseudomonadales*, *Xanthomonadales*, *Actinomycetales* and *Rhizobiales* collectively accounting for 32.7% of the dissimilarity (S4 Table.). The main distinguishing taxa associated with the sediment afters after 64 days were *Actinomycetales*, *Xanthomonadales*, *Spingomonadales*, *Rhodobacterales*, *Sphingobacteriales*, *Myxococcales*, *Nitrospirales*, *Gemmatales* and *Planctomycetales* (S5 Table.).

## Discussion

### Influence of artificial recharge on microbial abundance

Prior to the addition of synthetic wastewater, mean bacterial and VLP abundances from the column outflow were both in the range of 10^4^–10^7^ cells ml^-1^ (Fig 5). These counts are consistent with commonly reported microbial cell counts of 10^3^−10^8^ cells ml^-1^ in groundwater [25,44–48]. Following the addition of synthetic wastewater, mean bacterial and VLP abundances increased to 10^4^–10^8^ cells ml^-1^ (Fig 5). Several studies have shown that the productivity of microbial biofilms is distinctly higher in groundwater research areas where infiltrating surface waters lead to an increase in DOC supply [8,49–51]. Thus, the increased bacterial and VLP abundances in the water samples, and the subsequent decrease in flow rate in the columns following the addition of synthetic wastewater (Figs 4 and 5), is consistent with increased DOC concentrations promoting growth within the native microbial population (Fig 3). We suggest that this observed increase in microbial abundance combined with the subsequent reduction in flow rate may be indicative of microbial clogging within the column.

Previous studies have shown that aquifer recharge can significantly increase the abundance of microbial communities within days of injection [10]. In this study, mean VLP abundance increased over 2 orders of magnitude within 24 hours following the addition of synthetic wastewater (Fig 5B). We suggest that these viruses may have been attached to sediment particles or within bacterial cells, which were subsequently released following the addition of synthetic wastewater. The microbial communities forming biofilms in these systems therefore warrant further investigation. Furthermore, the synthetic wastewater used in this experiment was sterile, indicating that significant increases in abundances can occur without the introduction of additional cells and taxa. Therefore, we believe nutrient levels within recharge sources are to be assessed before injection to limit significant increases in microbial abundance, thereby reducing potential biofilm accumulation and clogging events.

The VBR has been used to investigate the relationship dynamics between viruses and bacteria in various aquatic systems, where high VBR values typify productive and nutrient-rich systems [52,53]. At day 0 we observed VBR values of approximately 10 and observed a decrease to 0.1–1.0 at day 34. Directly after the addition of synthetic wastewater on day 35, the VBR values increased up to 65.9 (Fig 5C). Previous research has shown that the VBR ratios of less than 10 indicate that conditions are unfavourable for viral mediated bacterial mortality, whereas VBR of greater than 10 corresponds to conditions that favour the lytic cycle [54,55]. The observed spike in the VBR may be directly related to the introduction of wastewater, whereby an increase in nutrient availability allows pent up bacteriophage reproduction causing extensive bacterial mortality, and thus altering the bacterial community composition. Rank abundance plots following nutrient addition (S1 Fig) further supports this theory, whereby previous research has shown that phage-bacterial kill-the-winner dynamics follow a power-law distribution [56]. Furthermore, a more negative exponent after the addition of synthetic wastewater indicates an increasing level of structural complexity [36,37]. Together, this suggests that following the addition of wastewater, viruses may be acting to structure the overall microbial community, causing major shifts in the bacterial taxa that dominate the system.

### Taxonomic profiling in column experiments

A shift in taxonomic composition was observed in the columns before and after the addition of synthetic wastewater, with fundamentally different communities inhabiting each phase of the experiment. Prior to the addition of synthetic wastewater, the main distinguishing taxa associated with the groundwater samples were *Sphingomonadales*, *Sphingobacteriales*, *Acidobacteriales*, *Rhodospirillales*, *Legionellales*, *Spartobacteriales*, *Syntrophobacterales*, *Bacillales*, and *Verrucomicrobiales*, collectively accounting for 35% of the dissimilarity (S4 Table.).

Aquifer systems are typically characterised as oligotrophic environments with low oxygen concentrations [57]. Consequently, the microbes typically inhabiting these environments are those that have adapted to such conditions. For example, *Sphingomonadales* are anaerobic, oligotrophic bacteria which are known to have a high degree of metabolic versatility [58,59], and as such were found to be an indicator taxa for freshwater aquifers [60]. Similarly, *Rhodospirillales* are in a South Australian unconfined aquifer [45], and are thus capable of adapting to groundwater ecosystems.

Following the addition of synthetic wastewater, the main distinguishing taxa were *Burkholderiales*, *Flavobacteriales*, *Pseudomonadales*, *Xanthomonadales*, *Actinomycetales* and *Rhizobiales*, collectively accounting for 32.7% of the dissimilarity (S4 Table.). A previous study compared the prevalence of pathogenic bacteria in flasks using groundwater or effluent flow through [61]. *Pseudomonas* sp. was found to persist in flasks where effluent was added and not in the flasks where groundwater was the main source. In our study, *Pseudomonadales* were present at low numbers prior to the addition of synthetic wastewater (0.3%), however became more dominant once nutrients were added (11.3%; S2 Table.). Thus, the addition of synthetic wastewater enhanced the occurrence of the pathogenic *Pseudomonadales* in the groundwater columns. Furthermore, as *Pseudomonadales* were not one of the main distinguishing taxa within the sediments of each column, these taxa appear to have remained planktonic in the water column (S5 Table.). Taken together, our data suggests that endemic pathogenic bacteria may be enhanced by the introduction of wastewater such as aquifer recharge and storage applications, and that planktonic as well as biofilm forming bacteria should be taken into account when assessing ASR site suitability.

Despite differences in nutrient concentrations and environmental conditions, substrate type ultimately structures microbial communities [45,62–64]. More recently, however, Smith et al. [65] showed that following a contamination event, substrate type becomes less important in structuring microbial communities. Following the addition of synthetic wastewater, *Pseudomonadales*, *Burkholderiales*, *Rhodobacterales*, dominated (S5 Table.). Furthermore, CAP analysis showed a significant difference between the groundwater samples before and after the addition of synthetic wastewater, with water samples exhibiting more similarity to sediment samples after wastewater addition (Fig 6). Collectively, these results suggest that the introduction of wastewater in ASR practices can fundamentally alter the taxonomic composition of the endemic microbial communities present, overriding any community structure present driven by substrate type.

Following the addition of synthetic wastewater, microbial diversity increased (S3 Fig). Cho and Kim [66] used restriction fragment length polymorphism patterns of cloned 16S rDNA libraries to show an increased diversity in subsurface aquifers receiving wastewater input. The authors concluded the increased diversity was due to the infiltrating wastewater. However, in our study the infiltrating wastewater was sterile and so the increased diversity is more likely accounted for by rare taxa increasing in abundance. This theory is supported by the rarefaction curves not reaching asymptotes (S3 Fig), which indicates that the rare taxa were not sampled.

Here we have demonstrated in a laboratory based experiment the effect wastewater addition has on the endemic groundwater microbial communities. Our data supports the need for flow cytometry and high-throughput sequencing approaches to capture the microbial community dynamics in groundwater ecosystems. Bar-coded pyrosequencing was implemented at 2 time points before and after the addition of synthetic wastewater to demonstrate for the first time the effect of wastewater on the community structure of the endemic groundwater microbial communities. The variations in community abundance throughout the 64 days however suggest that future studies should implement an increased sequencing effort to gain a more in depth description of community dynamics throughout. Fundamental shifts in microbial community dynamics and structure were seen within days of the wastewater addition. Furthermore, sterile wastewater was introduced to the column experiments indicating that endemic groundwater communities hold the potential to reduce flow rates in aquifers without the introduction of new taxa. Therefore, future field based studies on the effects of wastewater injection on natural groundwater ecosystems are warranted. Furthermore, our study suggests that a complete profile of the groundwater properties, including microbial community abundance and composition should be taken into consideration when selecting aquifers for ASR.

## Supporting Information

S1 Fig Log-log plot of rank versus cell concentration for (A) Mean bacterial and (B) Mean VLP over the 64 day experiment. Abundance counts before the addition of synthetic wastewater are represented in red, while abundance counts after the addition of synthetic wastewater are represented in blue. Power law trend lines were calculated with random noise omitted (grey points).
(TIF)

S2 Fig Phylum distribution of Bacterial 16S bar-coded pyrosequencing libraries. (TIF)

S3 Fig Rarefaction curves for bacterial communities within the column experiments. Each curve represents the overall, combined bacterial 16S rRNA metagenome recovered from each stage of the experiment. The rarefaction curve, plotting the Chao1 rarefaction measure as a function of the sequences per sample, was computed in QIIME. A) Orange represents groundwater, blue represents column groundwater before the addition of synthetic wastewater (day 35), red represents column groundwater after the addition of synthetic wastewater. B) blue represents sediment at 15 cm and red represents sediment at 0 cm.
(TIF)

S1 Table Relative proportion of phylum level matches to the ribosomal database project (RDP) database. (DOCX)

S2 Table Relative proportion of order level matches to the ribosomal database project (RDP) database. (DOCX)

S3 Table Results of CAP analysis (using *m* = 7 principal coordinate axes, explaining 99% of total variation) testing the hypothesis that the taxonomic composition differ before and after the addition of synthetic wastewater. Significant of trace and delta statistics was P = 0.0005 and P = 0.01, respectively and the first canonical axis alone explained 95% of the total variation.
(DOCX)

S4 Table Contribution of order level taxonomy to the dissimilarity of the groundwater samples before and after addition of synthetic wastewater. Average dissimilarity between the two groups is 55.7%. Only taxa that were consistent (i.e. Diss/SD > 1.4) are shown here. The larger value in each case (i.e. the potential indicator taxa) is shown in bold.
(DOCX)

S5 Table Contribution of order level taxonomy to the dissimilarity of the groundwater samples after the addition of synthetic wastewater and the sediment within the columns. Average dissimilarity between the two groups is 32.8%. Only taxa that were consistent (i.e. Diss/SD > 1.4) are shown here. The larger value in each case (i.e. the potential indicator taxa) is shown in bold.
(DOCX)
