# Supplementary figures and images for: Putative Effect of Aquifer Recharge on the Abundance and Taxonomic Composition of Endemic Microbial Communities
Source: PLoS One. 2015 Jun 17;10(6):e0129004. doi: 10.1371/journal.pone.0129004 (PMC4471229; doi:10.1371/journal.pone.0129004)

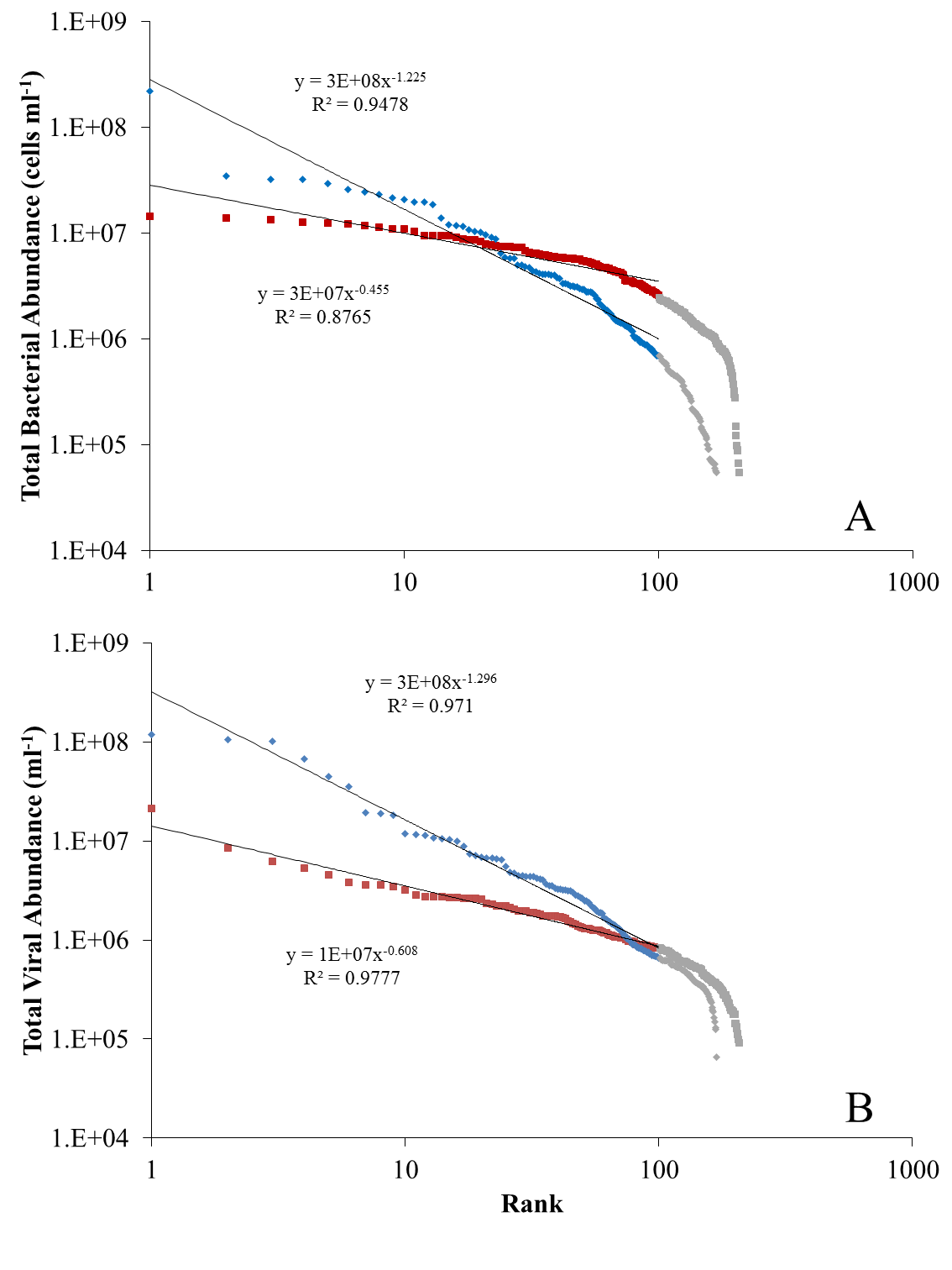

Supplement: S1 Fig — Abundance counts before the addition of synthetic wastewater are represented in red, while abundance counts after the addition of synthetic wastewater are represented in blue. Power law trend lines were calculated with random noise omitted (grey points). (TIF) [file pone.0129004.s001.tif]

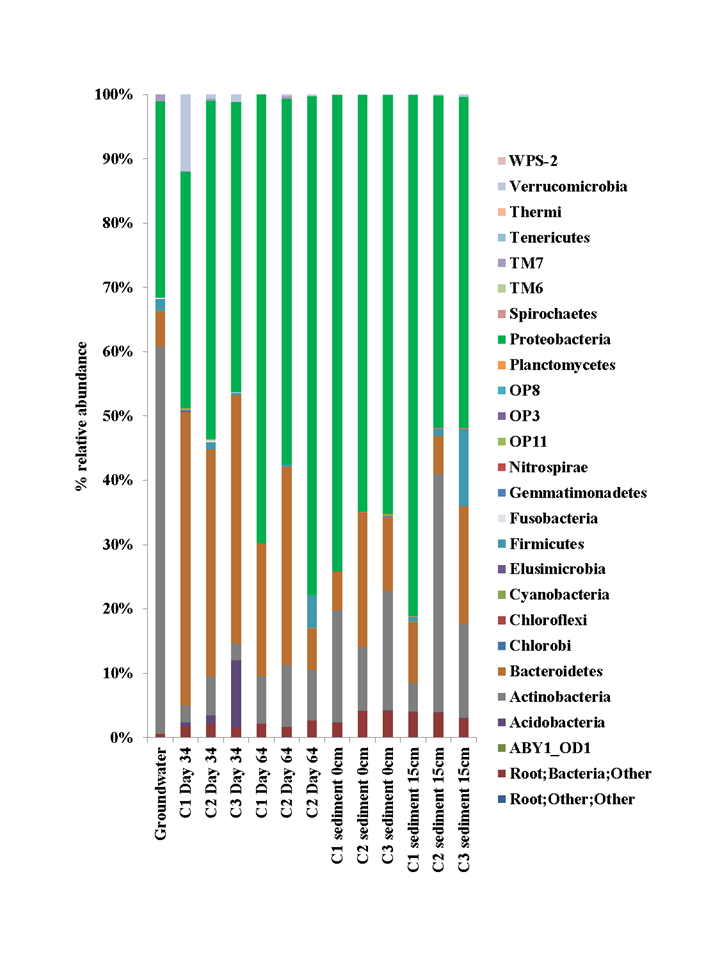

Supplement: S2 Fig — (TIF) [file pone.0129004.s002.tif]

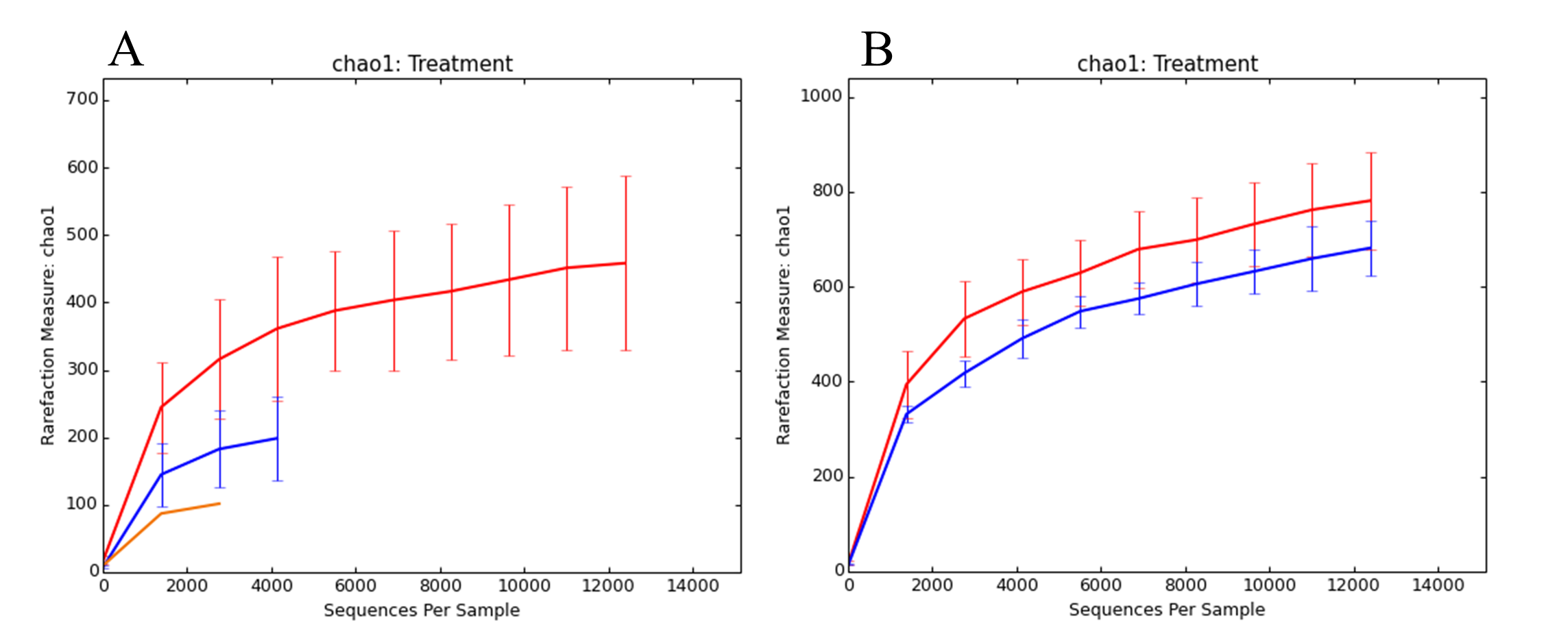

Supplement: S3 Fig — Each curve represents the overall, combined bacterial 16S rRNA metagenome recovered from each stage of the experiment. The rarefaction curve, plotting the Chao1 rarefaction measure as a function of the sequences per sample, was computed in QIIME. A) Orange represents groundwater, blue represents column groundwater before the addition of synthetic wastewater (day 35), red represents column groundwater after the addition of synthetic wastewater. B) blue represents sediment at 15 cm and red represents sediment at 0 cm. (TIF) [file pone.0129004.s003.tif]
