# Supplementary material for: Putative Effect of Aquifer Recharge on the Abundance and Taxonomic Composition of Endemic Microbial Communities
Source: PLoS One. 2015 Jun 17;10(6):e0129004. doi: 10.1371/journal.pone.0129004 (PMC4471229; doi:10.1371/journal.pone.0129004)
Supplement: S2 Table — (DOCX) [file pone.0129004.s005.docx]

**S2 Table.** Relative proportion of order level matches to the ribosomal database project (RDP) database.

| **Taxon** | **Ground**  **water** | **C1 Day 34** | **C2 Day 34** | **C3 Day 34** | **C1 Day 64** | **C2 Day 64** | **C3 Day 64** | **C1 sediment 0cm** | **C2 sediment 0cm** | **C3 sediment 0cm** | **C1 sediment 15cm** | **C2 sediment 15cm** | **C3 sediment 15cm** |
| --- | --- | --- | --- | --- | --- | --- | --- | --- | --- | --- | --- | --- | --- |
| **Other** | 0.000369481 | 0.000925 | 0.000479 | 9.60E-05 | 8.88E-05 | 0.000131 | 0.000333 | 0.00022315 | 0.000239971 | 0.000240778 | 0.000157911 | 9.48E-05 | 0.00017231 |
| **Bacteria (unknown phylum)** | 0.005726954 | 0.016615 | 0.02074 | 0.015655 | 0.021926 | 0.016278 | 0.026176 | 0.023016354 | 0.040755109 | 0.041702783 | 0.039267291 | 0.038654825 | 0.029249591 |
| **OD1 (unknown class)** | 0 | 2.72E-05 | 0 | 0 | 0 | 0 | 0 | 0 | 0 | 0 | 0 | 0 | 0 |
| ***Acidobacteriales*** | 0 | 0.005928 | 0.013401 | 0.103919 | 0 | 0 | 0 | 0 | 0 | 0 | 5.26E-05 | 0 | 4.31E-05 |
| ***Chloracidobacteria* (unknown order)** | 0 | 0.000408 | 0.000319 | 0 | 2.96E-05 | 0 | 0 | 0.00022315 | 0.000719914 | 0.000433401 | 0.000421097 | 0.000758557 | 0.000689239 |
| ***Holophagales*** | 0 | 0 | 0 | 0 | 0 | 0 | 0 | 0 | 0 | 0 | 0.000421097 | 0.000189639 | 0 |
| ***Solibacterales*** | 0 | 0 | 0 | 0 | 0 | 0 | 0 | 0 | 0 | 0 | 0 | 0 | 4.31E-05 |
| ***Actinobacteria* (unknown class)** | 0 | 0 | 0 | 0 | 0 | 0 | 4.75E-05 | 0.000191272 | 0.000119986 | 0.000192623 | 5.26E-05 | 0.000442492 | 0.000301542 |
| ***Actinobacteria* (Other)** | 0 | 0 | 0 | 0 | 0.000118 | 8.73E-05 | 9.50E-05 | 0.000350665 | 0.000159981 | 0.000337089 | 0.000105274 | 0.000347672 | 0.000215387 |
| ***Actinobacteria* (unknown order)** | 0 | 0 | 0 | 0 | 0 | 0 | 0 | 0 | 0 | 0 | 0 | 0 | 4.31E-05 |
| ***Acidimicrobiales*** | 0 | 0 | 0 | 0 | 0 | 4.36E-05 | 0 | 0 | 0 | 9.63E-05 | 0 | 0 | 0 |
| ***Actinomycetales*** | 0.59652688 | 0.026514 | 0.059828 | 0.025836 | 0.073529 | 0.096618 | 0.07886 | 0.173865919 | 0.099148102 | 0.185447366 | 0.044320455 | 0.368943393 | 0.146291031 |
| ***MC47*** | 0.004618511 | 0 | 0 | 0 | 0 | 0 | 0 | 0 | 0 | 0 | 0 | 0 | 0 |
| ***Solirubrobacterales*** | 0.001108443 | 0 | 0 | 0 | 0 | 0 | 0 | 0 | 0 | 0 | 0 | 0 | 0 |
| ***Bacteroidetes* (unknown class)** | 0.000369481 | 0.00068 | 0 | 9.60E-05 | 0.002841 | 0.003055 | 0.001235 | 0.000733208 | 0.004359477 | 0.003611673 | 0.00110538 | 0.000948197 | 0.000603084 |
| ***Bacteroidales*** | 0 | 5.44E-05 | 0.000479 | 0.000384 | 2.96E-05 | 0 | 9.50E-05 | 0 | 0 | 9.63E-05 | 0.000473734 | 0.000126426 | 0.00017231 |
| ***Flavobacteria* (unknown order)** | 0 | 0 | 0 | 0 | 0 | 0 | 0 | 0 | 4.00E-05 | 9.63E-05 | 0.000315823 | 0 | 8.62E-05 |
| ***Flavobacteriales*** | 0.021429891 | 0.000218 | 0.003031 | 0.000672 | 0.143834 | 0.249182 | 0.006651 | 0.021071759 | 0.185697716 | 0.017962053 | 0.017791346 | 0.020575872 | 0.028000345 |
| ***Sphingobacteriales*** | 0.033068539 | 0.454138 | 0.349713 | 0.385709 | 0.059682 | 0.055902 | 0.05677 | 0.037903663 | 0.01799784 | 0.091880959 | 0.075744815 | 0.037801448 | 0.153700353 |
| ***Ignavibacteriales*** | 0 | 0 | 0 | 0.000384 | 0 | 0 | 0 | 0 | 0 | 0 | 0 | 0 | 0 |
| ***ZB1*** | 0 | 0 | 0 | 0 | 0 | 0 | 0 | 0 | 0 | 0 | 0 | 0 | 4.31E-05 |
| ***Chloroflexi* (unknown class)** | 0 | 0.000109 | 0 | 0 | 0 | 0 | 0 | 0 | 0 | 0 | 0 | 0 | 0 |
| ***Anaerolineae* (unknown order)** | 0 | 0.000136 | 0 | 0 | 0 | 0 | 0 | 0 | 0 | 0 | 0 | 0 | 0 |
| ***SOGA31* (unknown order)** | 0 | 0 | 0 | 0 | 0 | 0 | 0 | 0 | 0 | 0 | 0 | 6.32E-05 | 0 |
| ***TK17* (unknown order)** | 0 | 0.001468 | 0 | 0.000192 | 0 | 0 | 0 | 0 | 0 | 0 | 0 | 0 | 0 |
| ***S085*** | 0.000369481 | 0 | 0 | 0 | 0 | 0 | 0 | 0 | 0 | 0 | 0 | 0 | 0 |
| ***mle1-48*** | 0 | 0 | 0 | 0 | 0 | 0 | 0 | 0 | 0 | 4.82E-05 | 0 | 3.16E-05 | 0 |
| ***Cyanobacteria* (unknown class)** | 0 | 0.000218 | 0 | 0 | 0 | 0 | 0 | 0 | 0 | 0 | 0 | 0 | 0 |
| ***SM1D11*** | 0 | 0 | 0 | 0 | 0 | 0 | 0 | 0 | 0 | 0 | 0 | 3.16E-05 | 0 |
| ***Stramenopiles*** | 0 | 0 | 0 | 0 | 0 | 0 | 0 | 0 | 0 | 0 | 5.26E-05 | 0 | 0 |
| ***Streptophyta*** | 0.000738962 | 0 | 0 | 0 | 0 | 0 | 0 | 0 | 0 | 0 | 0 | 0 | 0 |
| ***Synechococcales*** | 0 | 0 | 0 | 0 | 0 | 0 | 4.75E-05 | 0 | 0 | 0 | 0 | 0 | 0 |
| ***Elusimicrobiales*** | 0 | 0.000245 | 0 | 0 | 0 | 0 | 0 | 0 | 0 | 0 | 0 | 0 | 0 |
| ***Firmicutes* (unknown class)** | 0 | 0 | 0 | 0 | 0 | 0.000131 | 0.00114 | 0 | 0 | 0.000240778 | 0.000263186 | 0.000505705 | 0.001766176 |
| ***Bacilli* (Other)** | 0 | 0 | 0 | 0 | 0 | 8.73E-05 | 9.50E-05 | 0 | 0 | 0 | 0 | 6.32E-05 | 0.000560007 |
| ***Bacilli* (unknown order)** | 0 | 0 | 0 | 0 | 0 | 0 | 0.000238 | 0 | 0 | 0 | 0 | 0 | 0.000301542 |
| ***Bacillales*** | 0.008867541 | 0.000544 | 0.003191 | 0.003169 | 0 | 0.00048 | 4.75E-05 | 0 | 0 | 0 | 0 | 0 | 0 |
| ***Lactobacillales*** | 0.006465915 | 0.000843 | 0.005105 | 9.60E-05 | 0 | 0.000262 | 0.026936 | 0 | 4.00E-05 | 9.63E-05 | 0 | 0.000221246 | 0.103213578 |
| ***Clostridia* (unknown order)** | 0 | 0.000109 | 0 | 0 | 0 | 0 | 0 | 0 | 0 | 0 | 0 | 0 | 0 |
| ***Clostridiales*** | 0.002401626 | 0.000707 | 0.003031 | 0 | 2.96E-05 | 0.002138 | 0.022898 | 0 | 0 | 0.000866802 | 0.006790188 | 0.010177313 | 0.013483243 |
| ***Fusobacteriales*** | 0.002032145 | 0.000136 | 0.00335 | 9.60E-05 | 0 | 0 | 0 | 0 | 0 | 0 | 0 | 0 | 0 |
| ***Gemmatimonadales*** | 0 | 8.16E-05 | 0 | 0 | 0 | 0 | 0 | 0.000127514 | 0 | 0.000240778 | 0.000157911 | 9.48E-05 | 0.000560007 |
| ***Nitrospirales*** | 0 | 0.00019 | 0 | 9.60E-05 | 0 | 0 | 0 | 0.00022315 | 0.000279966 | 0.001589136 | 5.26E-05 | 0.000410885 | 0.000301542 |
| ***OP11-3* (unknown order)** | 0 | 0.000925 | 0.001755 | 0 | 0 | 8.73E-05 | 0.000143 | 0 | 0 | 0 | 0 | 0 | 0 |
| ***OP3* (unknown class)** | 0 | 5.44E-05 | 0 | 0 | 0 | 0 | 0 | 0 | 0 | 0 | 0 | 0 | 0 |
| ***GIF10*** | 0 | 2.72E-05 | 0 | 0 | 0 | 0 | 0 | 0 | 0 | 0 | 0 | 0 | 0 |
| ***OP8_1* (unknown order)** | 0 | 8.16E-05 | 0 | 9.60E-05 | 0 | 0 | 0 | 0 | 0 | 0 | 0 | 0 | 0 |
| ***Gemmatales*** | 0 | 0 | 0 | 0 | 0 | 0 | 4.75E-05 | 0.000286907 | 0.000599928 | 0.000963113 | 5.26E-05 | 0.000347672 | 0.000689239 |
| ***Pirellulales*** | 0 | 0 | 0 | 0 | 0 | 0 | 0 | 0 | 0 | 0 | 0 | 3.16E-05 | 0 |
| ***Planctomycetales*** | 0 | 0.000381 | 0 | 0.00048 | 0 | 0 | 0 | 6.38E-05 | 0.000159981 | 0.000240778 | 0.001000105 | 0.000347672 | 0.000258465 |
| ***CL500-15*** | 0 | 0 | 0 | 0 | 0 | 0 | 0 | 0 | 0 | 0 | 0 | 9.48E-05 | 0 |
| ***Proteobacteria* (unknown class)** | 0.000369481 | 0.007913 | 0.005265 | 0.003938 | 0.01151 | 0.008335 | 0.015914 | 0.014122223 | 0.024557053 | 0.02451122 | 0.014317297 | 0.01295869 | 0.009563195 |
| ***Alphaproteobacteria* (unknown order)** | 0.00018474 | 0.005983 | 0.004467 | 0.004226 | 0.000266 | 0.001004 | 0.004133 | 0.002454653 | 0.007239131 | 0.007656747 | 0.004263607 | 0.002654951 | 0.003015422 |
| ***Alphaproteobacteria* (unknown order)** | 0 | 8.16E-05 | 0 | 0 | 0 | 0 | 0 | 3.19E-05 | 0.000239971 | 0 | 0.000157911 | 0 | 0 |
| ***Caulobacterales*** | 0 | 0.015691 | 0.030313 | 0.015847 | 0.000207 | 0.005717 | 0.013254 | 0.001689566 | 0.004159501 | 0.006356544 | 0.001947573 | 0.003160656 | 0.003747738 |
| ***Rhizobiales*** | 0 | 0.000218 | 0 | 0 | 0.001006 | 0.002837 | 0.002803 | 0.001307023 | 0.002199736 | 0.00341905 | 0.003368776 | 0.002307279 | 0.002627725 |
| ***Rhodobacterales*** | 0 | 0.000598 | 0.000319 | 0 | 0.000355 | 0.001004 | 0.015392 | 0.008097166 | 0.033196016 | 0.019310411 | 0.016475418 | 0.008438952 | 0.012363229 |
| ***Rhodospirillales*** | 0.00018474 | 0.026052 | 0.015635 | 0.009988 | 0 | 0.000393 | 0.005273 | 0.00022315 | 0.000519938 | 0.000626023 | 0.001473839 | 0.001453902 | 0.00236926 |
| ***Rickettsiales*** | 0.047108812 | 0.000163 | 0.005743 | 0.001537 | 0 | 0.001047 | 0.00019 | 0.000318786 | 0.000119986 | 0.000337089 | 0.000105274 | 0 | 0 |
| ***Sphingomonadales*** | 0.001662664 | 0.179887 | 0.378271 | 0.361218 | 0.001065 | 0.012437 | 0.011164 | 0.018776499 | 0.019077711 | 0.032601368 | 0.042320244 | 0.029330889 | 0.023606444 |
| ***Betaproteobacteria* (unknown order)** | 0.002955847 | 0.000952 | 0.000479 | 9.60E-05 | 0.004054 | 0.00336 | 0.010641 | 0.008798495 | 0.009478863 | 0.014976404 | 0.017422887 | 0.010082493 | 0.008486258 |
| ***Burkholderiales*** | 0.160539442 | 0.091507 | 0.03813 | 0.022858 | 0.450556 | 0.343138 | 0.459145 | 0.588957251 | 0.239851218 | 0.307136666 | 0.145383725 | 0.059768008 | 0.113810632 |
| ***Gallionellales*** | 0 | 0.000245 | 0 | 0 | 0 | 0 | 4.75E-05 | 0 | 0 | 0 | 0 | 0 | 0 |
| ***Hydrogenophilales*** | 0 | 2.72E-05 | 0 | 0 | 0 | 0 | 9.50E-05 | 0 | 0 | 0.000144467 | 0 | 0 | 0 |
| ***Methylophilales*** | 0.004618511 | 0 | 0 | 9.60E-05 | 0 | 0 | 0 | 0 | 0.000199976 | 0.000288934 | 0 | 0.000158033 | 4.31E-05 |
| ***Neisseriales*** | 0 | 0.000707 | 0.001117 | 0.004706 | 2.96E-05 | 0.001789 | 0.088124 | 0.000286907 | 0.002839659 | 0.002359626 | 0.000105274 | 0.000189639 | 0.001809253 |
| ***Nitrosomonadales*** | 0 | 0 | 0 | 0 | 0 | 0 | 0 | 9.56E-05 | 0.000199976 | 0.000433401 | 5.26E-05 | 0 | 0.000258465 |
| ***Rhodocyclales*** | 0.015702937 | 0.006227 | 0.001755 | 0.000864 | 0.001657 | 0.008466 | 0.019002 | 0.017788262 | 0.032716074 | 0.066984494 | 0.524476261 | 0.308416827 | 0.231885931 |
| ***Deltaproteobacteria* (unknown order)** | 0 | 5.44E-05 | 0 | 0 | 0 | 0 | 0.000333 | 0 | 0 | 0 | 0.000105274 | 3.16E-05 | 0.001292324 |
| ***Bdellovibrionales*** | 0 | 0.000109 | 0.000479 | 0 | 0 | 0 | 0 | 0 | 0 | 0 | 0.000105274 | 0 | 0 |
| ***Desulfobacterales*** | 0 | 5.44E-05 | 0.00016 | 0.000192 | 0 | 4.36E-05 | 0.00019 | 0 | 0 | 0 | 0 | 0 | 0 |
| ***Desulfuromonadales*** | 0 | 0 | 0 | 0.004706 | 0 | 0 | 0 | 0 | 0 | 0 | 0 | 0 | 0 |
| ***Myxococcales*** | 0 | 0 | 0 | 9.60E-05 | 0 | 8.73E-05 | 0 | 0.000765087 | 0.001719794 | 0.002504093 | 0.000157911 | 0.000284459 | 0.000258465 |
| ***Syntrophobacterales*** | 0 | 0.000598 | 0.000798 | 0.005955 | 0 | 0 | 0 | 0 | 0 | 0 | 0 | 0 | 0 |
| ***Gammaproteobacteria* (unknown order)** | 0.001293183 | 0.002665 | 0.002553 | 0.000384 | 0.014824 | 0.012655 | 0.004038 | 0.007618987 | 0.026716794 | 0.016806318 | 0.002947679 | 0.006005247 | 0.002843112 |
| ***Aeromonadales*** | 0 | 0 | 0 | 0 | 0 | 0 | 9.50E-05 | 0 | 0 | 0 | 0 | 0 | 0.000258465 |
| ***Alteromonadales*** | 0 | 0 | 0 | 0 | 0 | 0.000175 | 0 | 0 | 0 | 0 | 0 | 0 | 0 |
| ***Chromatiales*** | 0 | 0.001659 | 0.000319 | 0 | 0 | 4.36E-05 | 0 | 3.19E-05 | 4.00E-05 | 0 | 5.26E-05 | 0.000189639 | 4.31E-05 |
| ***Enterobacteriales*** | 0.002216885 | 0 | 0 | 0 | 0 | 0.000218 | 0.003658 | 0 | 0 | 0 | 0 | 0 | 0.003230809 |
| ***Legionellales*** | 0 | 0.004161 | 0.015795 | 0.005955 | 0.000533 | 0.000175 | 0.000143 | 0.000988237 | 4.00E-05 | 9.63E-05 | 0.000526371 | 6.32E-05 | 4.31E-05 |
| ***Oceanospirillales*** | 0 | 0.00068 | 0.001914 | 0 | 5.92E-05 | 4.36E-05 | 9.50E-05 | 0 | 0 | 9.63E-05 | 0 | 0 | 0 |
| ***Pasteurellales*** | 0 | 0.00019 | 0.003669 | 0 | 0 | 4.36E-05 | 0 | 0 | 0 | 0 | 0 | 0 | 0 |
| ***Pseudomonadales*** | 0.055791613 | 0.003726 | 0.00351 | 0.000672 | 0.210291 | 0.07602 | 0.053302 | 0.062513947 | 0.147262329 | 0.076278532 | 0.029003053 | 0.027971807 | 0.005169294 |
| ***Thiotrichales*** | 0 | 0 | 0 | 0 | 2.96E-05 | 0.000262 | 0.000143 | 0.000255029 | 0.000559933 | 0.001011268 | 0.000421097 | 0.000600525 | 4.31E-05 |
| ***Xanthomonadales*** | 0.01256235 | 0.018383 | 0.015635 | 0.007876 | 0.001391 | 0.089286 | 0.068124 | 0.00618445 | 0.096188457 | 0.069055186 | 0.00547426 | 0.04311135 | 0.088739554 |
| ***Leptospirales*** | 0 | 0 | 0 | 0 | 0 | 0 | 0 | 0 | 0 | 4.82E-05 | 0 | 0 | 0 |
| ***SJA-4* (unknown order)** | 0 | 0 | 0 | 0 | 0 | 8.73E-05 | 0 | 0 | 8.00E-05 | 4.82E-05 | 0 | 0 | 0 |
| ***TM7-1* (unknown order)** | 0 | 8.16E-05 | 0.00016 | 0 | 0 | 0 | 0 | 0 | 0 | 0 | 0 | 0 | 0 |
| ***TM7-3* (unknown order)** | 0 | 2.72E-05 | 0.000479 | 9.60E-05 | 0 | 0.000655 | 4.75E-05 | 0 | 0 | 0 | 0 | 0 | 0 |
| ***EW055*** | 0.010530205 | 0.000109 | 0.002074 | 0 | 0 | 0.003229 | 0 | 0 | 0 | 0 | 0 | 0 | 0 |
| ***I025*** | 0 | 0 | 0.000319 | 9.60E-05 | 0 | 8.73E-05 | 0 | 0 | 0 | 0 | 0 | 0 | 0 |
| ***Erysipelotrichales*** | 0 | 0 | 0 | 0.000384 | 0 | 0 | 0 | 0 | 0 | 0 | 0 | 0 | 8.62E-05 |
| ***RF39*** | 0 | 0 | 0.00016 | 0 | 0 | 0 | 0 | 0 | 0 | 0 | 0 | 0 | 0 |
| ***Thermales*** | 0.00018474 | 0 | 0 | 0 | 0 | 0 | 0 | 0 | 0 | 0 | 0 | 0 | 0 |
| ***Verrucomicrobia* (unknown class)** | 0 | 0.000897 | 0.00016 | 0 | 0 | 0 | 0 | 0 | 0 | 0 | 0 | 0 | 0 |
| ***Opitutae* (Other)** | 0 | 0.001713 | 0 | 0.000288 | 0 | 0 | 4.75E-05 | 0 | 0 | 0 | 0 | 0 | 0 |
| ***Opitutae* (unknown order)** | 0 | 0.000136 | 0 | 9.60E-05 | 0 | 0 | 0 | 0 | 0 | 0 | 0 | 0 | 0 |
| ***Opitutales*** | 0 | 0.104017 | 0.002872 | 0.010085 | 0 | 0.002662 | 0.00266 | 0.000127514 | 0.000239971 | 0.000481556 | 0.00073692 | 0.001390689 | 0.003575429 |
| ***Spartobacteriales*** | 0 | 0.011122 | 0.001755 | 0.000576 | 2.96E-05 | 0 | 0 | 0 | 0 | 0 | 0 | 0 | 0 |
| ***Verrucomicrobiales*** | 0 | 0.001604 | 0.001276 | 0.000192 | 0 | 0 | 4.75E-05 | 0 | 8.00E-05 | 4.82E-05 | 0 | 0 | 4.31E-05 |
| ***WPS-2* (unknown class)** | 0 | 0 | 0 | 0 | 2.96E-05 | 0.000218 | 4.75E-05 | 0.000286907 | 0.000159981 | 0 | 0 | 0.000126426 | 0 |
