# Supplementary material for: Putative Effect of Aquifer Recharge on the Abundance and Taxonomic Composition of Endemic Microbial Communities
Source: PLoS One. 2015 Jun 17;10(6):e0129004. doi: 10.1371/journal.pone.0129004 (PMC4471229; doi:10.1371/journal.pone.0129004)
Supplement: S3 Table — Significant of trace and delta statistics was P = 0.0005 and P = 0.01, respectively and the first canonical axis alone explained 95% of the total variation. (DOCX) [file pone.0129004.s006.docx]

**S3 Table**. Results of CAP analysis (using *m* = 7 principal coordinate axes, explaining 99 % of total variation) testing the hypothesis that the taxonomic composition differ before and after the addition of synthetic wastewater. Significant of trace and delta statistics was P = 0.0005 and P = 0.01, respectively and the first canonical axis alone explained 95 % of the total variation.

| **Contaminant**  **Result** | **Groundwater** | **Before synthetic wastewater** | **After synthetic wastewater** | **sediment** | **Total** |
| --- | --- | --- | --- | --- | --- |
| **Allocation Success %** | 100 | 100 | 100 | 100 | 100 |
| **Ratio of correct:total** | 1:1 | 3:3 | 3:3 | 6:6 | 13:13 |
