# Supplementary material for: Putative Effect of Aquifer Recharge on the Abundance and Taxonomic Composition of Endemic Microbial Communities
Source: PLoS One. 2015 Jun 17;10(6):e0129004. doi: 10.1371/journal.pone.0129004 (PMC4471229; doi:10.1371/journal.pone.0129004)
Supplement: S4 Table — Average dissimilarity between the two groups is 55.7%. Only taxa that were consistent (i.e. Diss/SD > 1.4) are shown here. The larger value in each case (i.e. the potential indicator taxa) is shown in bold. (DOCX) [file pone.0129004.s007.docx]

**S4 Table.** Contribution of order level taxonomy to the dissimilarity of the groundwater samples before and after addition of synthetic wastewater. Average dissimilarity between the two groups is 55.7 %. Only taxa that were consistent (i.e. Diss/SD > 1.4) are shown here. The larger value in each case (i.e. the potential indicator taxa) is shown in bold.

|  | **Average Abundance** | |  |  |
| --- | --- | --- | --- | --- |
| **Taxonomy** | **Groundwater Day 34** | **Groundwater Day 64** | **Diss/**  **SD** | **Cum%** |
| *Sphingomonadales* | **0.55** | 0.08 | 3.73 | 11.97 |
| *Burkholderiales* | 0.22 | **0.64** | 4.29 | 23 |
| *Sphingobacteriales* | **0.63** | 0.24 | 9.32 | 32.95 |
| *Flavobacteriales* | 0.03 | **0.32** | 1.54 | 40.49 |
| *Pseudomonadales* | 0.05 | **0.32** | 2.13 | 47.66 |
| *Acidobacteriales* | **0.17** | 0 | 1.41 | 52.16 |
| *Xanthomonadales* | 0.12 | **0.2** | 2.82 | 55.57 |
| *Rhodospirillales* | **0.13** | 0.03 | 2.19 | 58.11 |
| *Actinomycetales* | 0.19 | **0.29** | 2.15 | 60.62 |
| *Legionellales* | **0.09** | 0.02 | 2.66 | 62.48 |
| *Gammaproteobacteria* (unknown order) | 0.04 | **0.1** | 1.71 | 64.03 |
| *Spartobacteriales* | **0.06** | 0 | 1.53 | 65.42 |
| *Betaproteobacteria* (unknown order) | 0.02 | **0.07** | 2.52 | 66.79 |
| *Syntrophobacterales* | **0.04** | 0 | 1.58 | 67.92 |
| *Rhizobiales* | 0 | **0.05** | 3.43 | 68.96 |
| *Bacteroidetes* (unknown class) | 0.01 | **0.05** | 2.26 | 69.89 |
| *Bacillales* | **0.05** | 0.01 | 1.75 | 70.82 |
| *Proteobacteria* (unknown class) | 0.07 | **0.11** | 1.79 | 71.68 |
| *Alphaproteobacteria* (unknown order) | **0.07** | 0.04 | 1.41 | 72.54 |
| *Verrucomicrobiales* | **0.03** | 0 | 2.2 | 73.24 |

Diss=dissimilarity; SD=Standard Deviation; Cum %=cumulative percentage of contribution to overall dissimilarity, Average Abundance values are reported for square-root transformed data
