# Supplementary material for: Putative Effect of Aquifer Recharge on the Abundance and Taxonomic Composition of Endemic Microbial Communities
Source: PLoS One. 2015 Jun 17;10(6):e0129004. doi: 10.1371/journal.pone.0129004 (PMC4471229; doi:10.1371/journal.pone.0129004)
Supplement: S5 Table — Average dissimilarity between the two groups is 32.8%. Only taxa that were consistent (i.e. Diss/SD > 1.4) are shown here. The larger value in each case (i.e. the potential indicator taxa) is shown in bold. (DOCX) [file pone.0129004.s008.docx]

**S5 Table.** Contribution of order level taxonomy to the dissimilarity of the groundwater samples after the addition of synthetic wastewater and the sediment within the columns. Average dissimilarity between the two groups is 32.8 %. Only taxa that were consistent (i.e. Diss/SD > 1.4) are shown here. The larger value in each case (i.e. the potential indicator taxa) is shown in bold.

|  | **Average Abundance** | |  |  |
| --- | --- | --- | --- | --- |
| **Taxonomy** | **Groundwater Day 64** | **Sediment Day 64** | **Diss/**  **SD** | **Cum%** |
| *Burkholderiales* | **0.64** | 0.46 | 1.82 | 9.64 |
| *Flavobacteriales* | **0.32** | 0.19 | 1.62 | 18.68 |
| *Sphingomonadales* | 0.08 | **0.16** | 1.64 | 22.28 |
| *Rhodobacterales* | 0.06 | **0.12** | 1.6 | 25.56 |
| *Sphingobacteriales* | 0.24 | **0.25** | 1.83 | 28.72 |
| *Clostridiales* | **0.07** | 0.05 | 1.44 | 31.48 |
| *Bacteria* (unknown phylum) | 0.15 | **0.19** | 1.89 | 33.31 |
| *Gammaproteobacteria* (unknown order) | **0.1** | 0.09 | 1.63 | 35.07 |
| *Caulobacterales* | **0.07** | 0.06 | 1.98 | 36.79 |
| *Betaproteobacteria* (unknown order) | 0.07 | **0.11** | 1.59 | 38.31 |
| *Alphaproteobacteria* | 0.04 | **0.07** | 1.53 | 39.73 |
| *Rhodospirillales* | 0.03 | 0.03 | 1.77 | 40.96 |
| *Opitutales* | 0.03 | 0.03 | 1.9 | 42.11 |
| *Myxococcales* | 0 | **0.03** | 1.65 | 43.16 |
| *Chloracidobacteria* (unknown order) | 0 | **0.02** | 4.68 | 44.05 |
| *Nitrospirales* | 0 | **0.02** | 1.97 | 44.88 |
| *Gemmatales* | 0 | **0.02** | 2.19 | 45.67 |
| *Bacteroidetes* (unknown class) | **0.05** | 0.04 | 1.73 | 46.44 |
| *Planctomycetales* | 0 | **0.02** | 2.17 | 47.18 |
| *Gemmatimonadales* | 0 | **0.01** | 1.74 | 47.7 |

Diss=dissimilarity; SD=Standard Deviation; Cum %=cumulative percentage of contribution to overall dissimilarity, Average Abundance values are reported for square-root transformed data
